# Efficacy of brain-computer interface training with motor imagery-contingent feedback in improving upper limb function and neuroplasticity among persons with chronic stroke: a double-blinded, parallel-group, randomized controlled trial

**DOI:** 10.1186/s12984-024-01535-2

**Published:** 2025-01-06

**Authors:** Myeong Sun Kim, Hyunju Park, Ilho Kwon, Kwang-Ok An, Hayeon Kim, Gyulee Park, Wooseok Hyung, Chang-Hwan Im, Joon-Ho Shin

**Affiliations:** 1 Translational Research Center for Rehabilitation Robots, National Rehabilitation Center, Ministry of Health and Welfare, Seoul, Korea; 2 Department of Rehabilitative and Assistive Technology, Rehabilitation Research Institute, National Rehabilitation Center, Ministry of Health and Welfare, Seoul, Korea; 3 Department of Healthcare and Public Health Research, Rehabilitation Research Institute, National Rehabilitation Center, Ministry of Health and Welfare, Seoul, Korea; 4 Department of Artificial Intelligence, Hanyang University, Seoul, Republic of Korea; 5 Department of Biomedical Engineering, Hanyang University, Seoul, Republic of Korea; 6 Department of Rehabilitation Medicine, National Rehabilitation Center, Ministry of Health and Welfare, Seoul, Korea; 7 Department of Rehabilitation Medicine, National Rehabilitation Center, Seoul, 01022 Korea

**Keywords:** Stroke, Rehabilitation, Brain-machine interface, Brain-computer interface, Randomized clinical trial

## Abstract

**Background:**

Brain-computer interface (BCI) technology can enhance neural plasticity and motor recovery in persons with stroke. However, the effects of BCI training with motor imagery (MI)-contingent feedback versus MI-independent feedback remain unclear. This study aimed to investigate whether the contingent connection between MI-induced brain activity and feedback influences functional and neural plasticity outcomes. We hypothesized that BCI training, with MI-contingent feedback, would result in greater improvements in upper limb function and neural plasticity compared to BCI training, with MI-independent feedback.

**Methods:**

This randomized controlled trial included persons with chronic stroke who underwent BCI training involving functional electrical stimulation feedback on the affected wrist extensor. Primary outcomes included the Medical Research Council (MRC) scale score for muscle strength in the wrist extensor (MRC-WE) and active range of motion in wrist extension (AROM-WE). Resting-state electroencephalogram recordings were used to assess neural plasticity.

**Results:**

Compared to the MI-independent feedback BCI group, the MI-contingent feedback BCI group showed significantly greater improvements in MRC-WE scores (mean difference = 0.52, 95% CI = 0.03–1.00, *p* = 0.036) and demonstrated increased AROM-WE at 4 weeks post-intervention (*p* = 0.019). Enhanced functional connectivity in the affected hemisphere was observed in the MI-contingent feedback BCI group, correlating with MRC-WE and Fugl-Meyer assessment-distal scores. Improvements were also observed in the unaffected hemisphere’s functional connectivity.

**Conclusions:**

BCI training with MI-contingent feedback is more effective than MI-independent feedback in improving AROM-WE, MRC, and neural plasticity in individuals with chronic stroke. BCI technology could be a valuable addition to conventional rehabilitation for stroke survivors, enhancing recovery outcomes.

**Trial registration:**

CRIS (KCT0009013).

**Supplementary Information:**

The online version contains supplementary material available at 10.1186/s12984-024-01535-2.

## Background

Upper limb impairments, which are common after stroke, have a significant impact on stroke survivors’ lives. Recent advancements in technologies, such as virtual rehabilitation, rehabilitation robots, and non-invasive brain stimulation, have enabled their use as adjunct or stand-alone therapies for upper-limb rehabilitation [1]. More recently, a brain-computer interface (BCI) system, that captures central nervous system (CNS) activity and translates it into artificial signals, has been used to substitute, restore, or enhance CNS output [2]. BCI allows direct communication between the human brain and external devices, enabling control of external devices, such as computer or robotic devices, bypassing conventional motor pathways. In upper-limb rehabilitation among persons with stroke, BCIs interpret the patient’s intention to move, aiding muscle stimulation or external device control. Through repetitive learning, BCIs can facilitate neural plasticity and fundamental motor recovery [3]. Several studies have demonstrated the beneficial effects of BCI training on motor function and neuroplasticity during stroke rehabilitation [4, 5].

A BCI system continuously monitors brain signals and provides feedback or stimulation to the user based on brain signals across various processes such as data acquisition, signal processing, feedback, adaptive training, and progress monitoring [4–6]. In the context of motor rehabilitation, reward feedback is provided only when the user imagines the desired movement, allowing the user to learn how to control the movement more effectively. The patient’s intention-driven feedback gradually creates a closed loop from intention to motor execution throughout BCI training, becoming an integral part of motor learning. Therefore, a contingency between the neural correlates of motor intention and consequent feedback should be established in BCIs to reorganize the targeted neural circuit, fundamentally leading to functional improvement.

Previous studies have demonstrated the favorable effects of this close connection between intention and feedback; however, there are inconsistencies in BCI systems and results of previous studies comparing motor imagery (MI)-contingent feedback (real-BCI) and BCI operated by MI-independent feedback (sham-BCI). Frolov et al. [7] employed a BCI-controlled hand exoskeleton and demonstrated within-group improvements after real-BCI without directly comparing real-BCI and sham-BCI. Ramos-Murguialday et al. [8] compared real and sham-BCI using BCI-driven finger orthosis and demonstrated significant improvement in motor function, particularly in terms of the upper limb Fugl-Meyer assessment (FMA) scores in the real-BCI group compared to those in the sham-BCI group. In addition, the improvements were associated with changes in the affected hand’s fMRI laterality index and electromyographic activity. Biasiucci et al. [9] compared real and sham-BCI using functional electrical stimulation (FES) feedback and demonstrated significant differences in the improvement of FMA, muscle strength of the wrist extensor, and functional connectivity in the affected hemisphere in the real-BCI group compared with that in the sham-BCI group.

recoveriX-PRO^®^ (g.tec Medical Engineering GmbH, Austria) is a ready-to-use BCI system and comprises different features to strengthen closed-loops. First, it detects motor intention in different ways. recoveriX-PRO compares brain activity between hemispheres during mental rehearsal of affected or unaffected (right or left) hand movements. This approach differs from previous methods that obtained signals from MI of the affected hand versus rest. Second, calibration is conducted in every session before the BCI intervention, reflecting the variability of electrode position and electroencephalogram (EEG) electrode impedance. Third, FES is provided during calibration, aiming to align more closely with motor intentions during BCI training, as EEG signals are influenced by sensory feedback during actual BCI-FES training. Lastly, recoveriX-PRO provides visual feedback through animated upper extremities of an avatar in virtual reality and proprioceptive feedback by generating movement via FES. In contrast to traditional BCIs, our study used a virtual reality-based game task. We believe that virtual reality enhances motor performance by boosting motivation and active engagement, which facilitate BCI participation [10]. We hypothesized that close contingent connection between MI-induced brain activity and consequent sensory feedback is essential in BCI systems for functional improvement and neural plasticity and that this contingency should be confirmed for individual BCI systems, considering their unique characteristics. Therefore, this study aimed to compare the effects of the BCI system operated by MI-contingent feedback BCI group, versus the effects of BCI operated by MI-independent feedback BCI group on distal upper limb function and brain activity in persons with chronic stroke with weak wrist extensor strength.

## Methods

### Study design

**Fig. 1:**
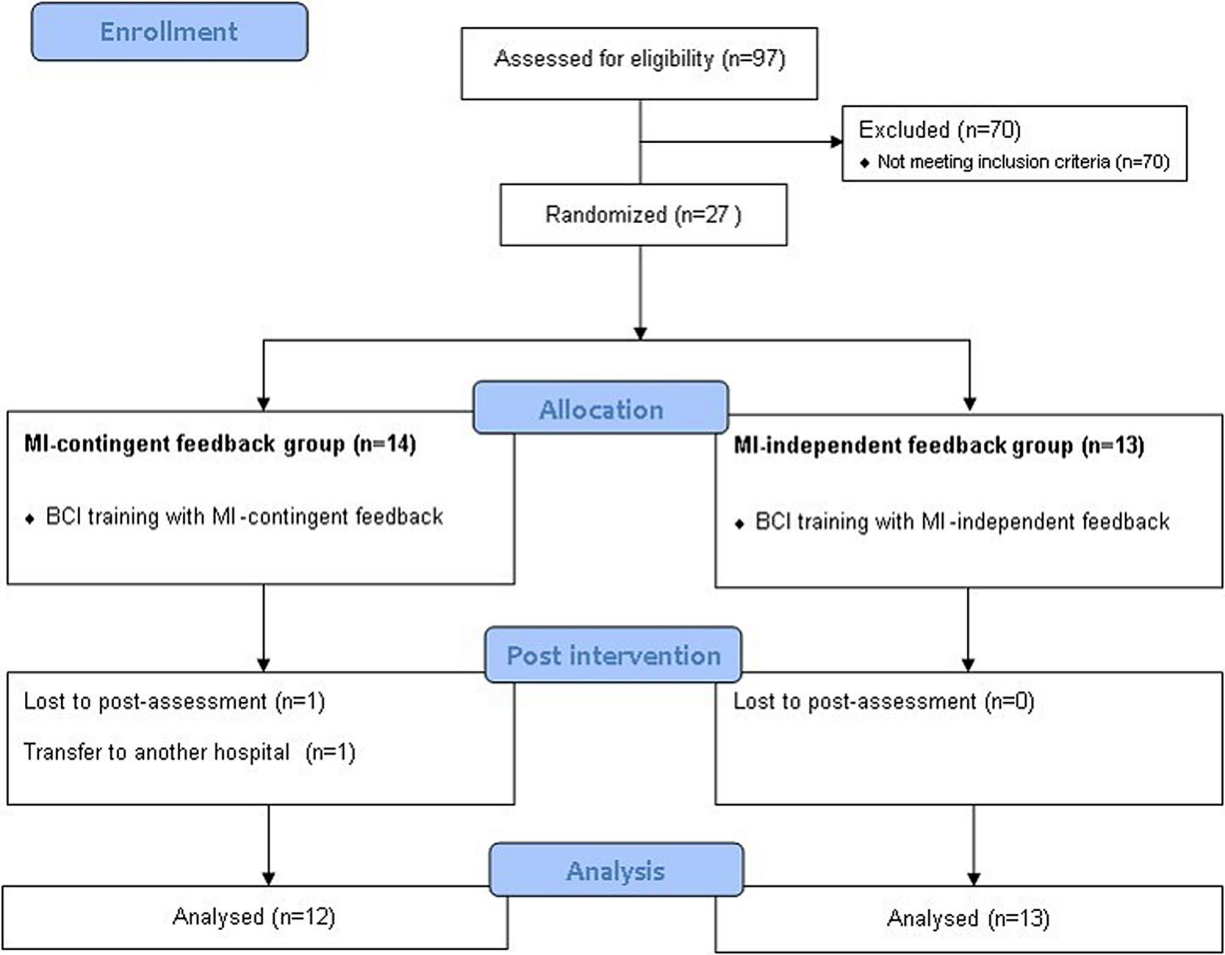
CONSORT flow diagram of participant recruitment

This double-blinded, parallel-group, randomized controlled trial was performed at a single rehabilitation hospital from August 2020 to December 2022. A computer-generated randomization table randomly allocated participants to the MI-contingent feedback BCI group or MI-independent feedback BCI group in a 1:1 ratio. The participants and assessors were blinded to the groups to which the participants were allocated. A CONSORT diagram is shown in Fig. 1. This study was approved by the Institutional Review Board of the Rehabilitation Hospital (NRC-2020-01-007) and registered at CRIS (KCT0009013). Participants provided informed consent before enrolment in the study.

### Participants

Participants were recruited from a rehabilitation hospital, adhering to specific inclusion and exclusion criteria. Inclusion criteria comprised individuals who: (1) had hemiplegia due to a first-ever stroke with unilateral hemisphere lesions; (2) were in the chronic stage of stroke (≥ 6 months post-onset); (3) exhibited Medical Research Council (MRC) scale scores indicating affected wrist extensor muscle weakness (≤ 2); and (4) were aged > 19 years. Exclusion criteria included: (1) conditions hindering EEG signal recording, such as scalp wounds or metal implants; (2) wrist flexor spasticity rated ≥ 2 on the modified Ashworth scale (MAS); (3) cognitive impairments or aphasia affecting comprehension; (4) neurological or psychiatric disorders unrelated to stroke; (5) musculoskeletal issues or severe pain in the affected upper limb affecting intervention; (6) hemispatial neglect; and (7) uncontrolled medical conditions.

Given the lack of prior reports on the differential effects of MI-contingent feedback and MI-independent feedback with identical BCI systems, determining a precise sample size beforehand was unfeasible. Consequently, we set a sample size of 12 participants per arm, totaling 24, which met the minimum recommended sample size for pilot trials. To account for a potential attrition rate of 10%, we targeted 27 participants for this pilot study [11].

### BCI-FES system

The BCI system utilized in this study was the recoveriX-PRO, a non-invasive, neurofeedback-based stroke rehabilitation system. The recoveriX-PRO comprises an EEG, FES, and a computer screen projecting virtual hands. Sixteen channels (FC3, FCz, FC4, C5, C3, C1, Cz, C2, C4, C6, CP3, CP1, CPz, CP2, CP4, and Pz of the international 10–20 systems) of the EEG signal recording system were employed, sampling at 256 Hz and digitally filtered with a 0.5–30 Hz bandpass filter. The ground and reference electrodes were positioned over the forehead (FPz) and right earlobe, respectively. Spatial filtering was conducted using the common spatial pattern method to optimize variance for one MI category while minimizing it for the other. Subsequently, the spatially filtered data underwent classification through linear discriminant analysis. A comprehensive description of the signal processing methods can be found elsewhere [6].

**Fig. 2:**
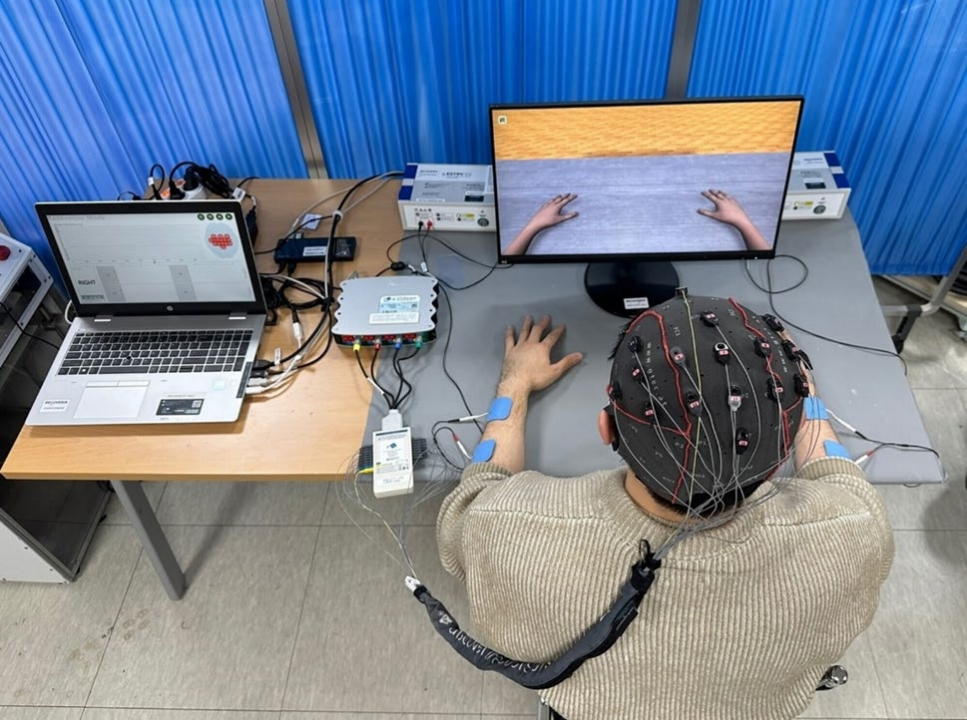
The recoveriX PRO training system

One pair of FES electrodes was positioned on the skin over both forearms’ wrist extensors. The frequency of the FES devices (g.Estim FES, g.tec Medical Engineering GmbH, Austria) was set at 50 Hz, with a rectangular pulse width of 300 µs. The current amplitude was adjusted individually to ensure contraction of the affected wrist extensor without causing discomfort. Visual feedback was provided in the form of an embodied representation, where the bilateral forearms and hands of a virtual avatar were displayed on a monitor. The recoveriX-PRO system seamlessly acquired, analyzed, and interpreted EEG signals associated with MI. It then activated the FES system when the participant imagined movement on the instructed side (Fig. 2). Utilizing the recoveriX-PRO system involves integrating cortical and peripheral activities, thereby establishing a closed loop between brain signals during imagined movements and contingent visual and proprioceptive feedback. This process aids patients in learning to imagine or execute desired movements effectively.

### BCI intervention

**Fig. 3:**
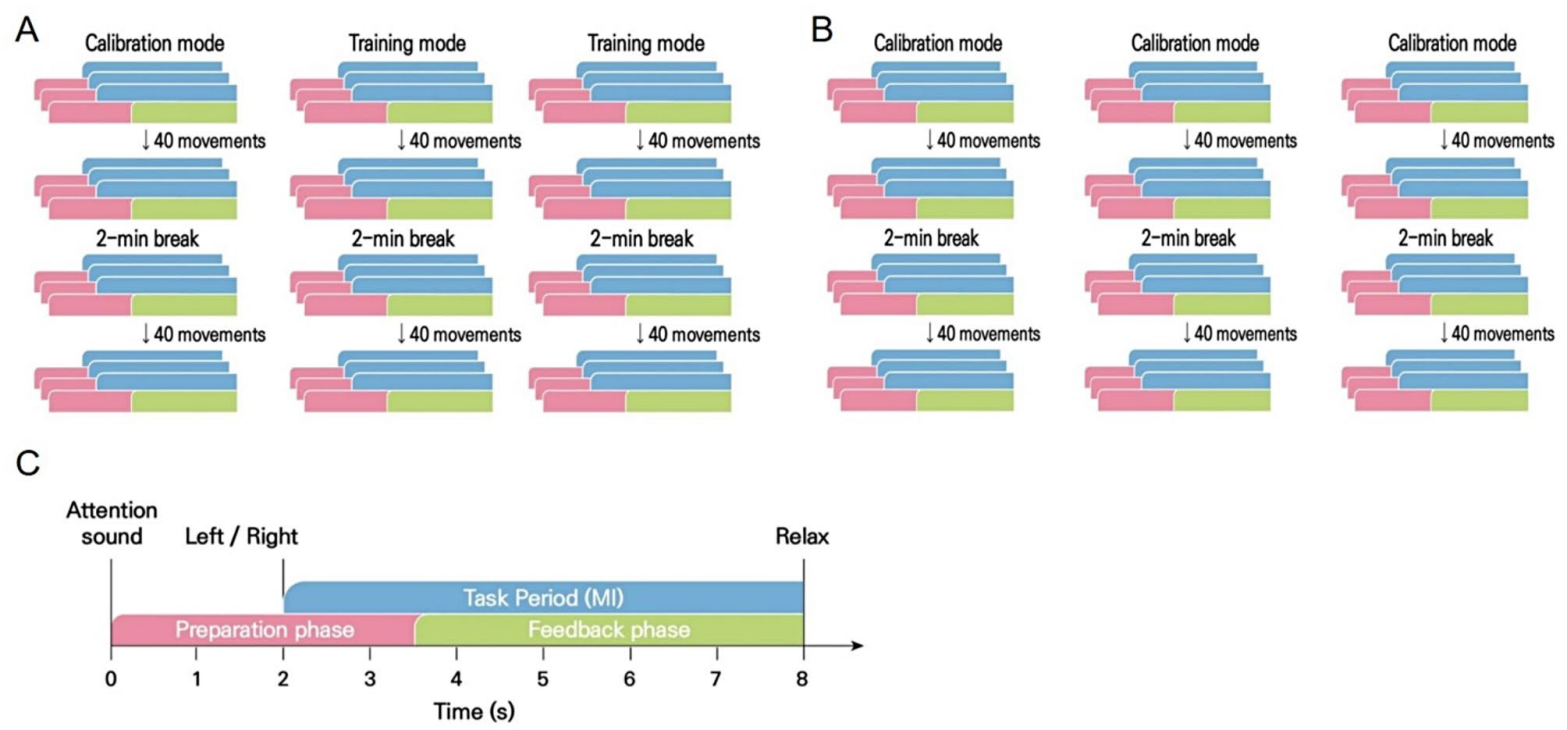
The recoveriX PRO session

The recoveriX-PRO interventions consisted of 240 trials of MI tasks involving both hands, divided into three runs of 80 trials each (refer to Fig. 3). Each run comprised two sets of 40 trials, separated by a 2-min break. An additional 3–5 min were allotted for the inter-run break. A trial commenced with an attention beep at 0 s. Subsequently, at 2 s, an arrow indicating the hand (“left” or “right”) for which MI was expected appeared on the monitor, accompanied by verbal instructions. Participants were instructed to imagine wrist dorsiflexion according to the system’s instruction, which alternated between “left” and “right” in a semi-random order. During the feedback phase (from 3.5 s to 8 s), FES and the virtual avatar were activated. The recoveriX-PRO interventions encompass two types of runs: calibration and rehabilitation runs. In the rehabilitation run, feedback was triggered only when the BCI system detected MI of the correct hand (MI-contingent feedback). Conversely, in the calibration run, feedback was consistently activated, irrespective of MI (MI-independent feedback). The feedback was updated at a rate of five times per second.

At 8 s, a relaxation signal indicated the end of the task period (MI), which lasted 6 s. The interval between trials varied randomly within a range of 1 s.

All participants underwent 20 sessions of 60-min BCI intervention, administered by research physical therapists, 5 days a week over 4 weeks. The BCI intervention session comprised one calibration run followed by two rehabilitation runs, where participants received MI-contingent feedback-based BCI intervention (Fig. 3A). In contrast, the MI-independent feedback group intervention session involved three consecutive calibration runs without any rehabilitation runs, providing participants with MI-independent feedback irrespective of their MI (Fig. 3B). Patients in the MI-independent feedback group used the same hardware, followed the same task instructions, and performed the same MI task of extending the affected wrist as those in the BCI group. Both groups had the same setup, with interventions starting based on the initial Calibration MI. The MI-contingent feedback group received FES only if it matched the Calibration MI, while the MI-independent feedback group received FES regardless of MI. Participants performed the MI task while seated, wearing the EEG cap, and observing the virtual avatar’s forearm and hand on a screen. The intervention and assessments were conducted in a dedicated, tranquil research room to facilitate task concentration. Additionally, all participants received 30 min of conventional therapy for the affected upper limb 5 days a week.

### Outcome assessment

Clinical and neurophysiological outcomes were evaluated by an independent team comprising a research physical therapist and a physiatrist who were blinded to the group allocation. Clinical assessments were conducted at baseline (W0), after 2 weeks (W2), and at the end of the 4-week intervention period (W4), while neurophysiological outcomes were recorded at W0 and W4.

### Clinical outcomes

We assessed baseline demographics of participants, including sex, age, stroke type, hemiparetic side, and time since stroke onset. Our evaluation aimed to cover all domains of upper limb function outlined in the International Classification of Functioning, Disability, and Health (ICF): impairment, activity, and participation. For body function/structure, we recorded outcomes such as the MRC scale for muscle strength in the wrist extensor (MRC-WE; scored 0–5), active range of motion in wrist extension (AROM-WE), and MAS for wrist flexor spasticity (MAS-WF; scored 0–4), along with the FMA. Activity and participation were assessed using the Box and Block Test (BBT) and the Stroke Impact Scale (SIS), respectively [12]. The primary outcome was changes in the MRC-WE and AROM-WE at W4, the targeted outcomes of our intervention.

The FMA tool is used to evaluate motor performance in persons with stroke, with higher scores indicating better motor function [13, 14]. We examined four specific FMA variables: FMA-total (0–66), FMA-distal (0–24), FMA-wrist (0–10), and FMA-hand (0–14). The BBT measures gross manual dexterity by counting the number of blocks an individual can transfer between sections within one minute. To assess health-related quality of life (HRQoL), we utilized version 3.0 of the SIS, a self-reported questionnaire tailored for persons with stroke. We focused on SIS domains relevant to upper limb function: hand function, physical and instrumental activities of daily living (ADLs/IADLs), and social participation [15, 16]. MI accuracy is an indicator of how well a participant is adapting to a BCI system. Each participant underwent 20 sessions, and MI accuracy was recorded in each session. The mean MI accuracy over the 20 sessions was then calculated to determine the mean MI accuracy for each group.

### Resting-state EEG measurements

Resting-state EEG data were collected to assess changes in functional connectivity in the motor area pre- and post-intervention. EEG recordings took place during separate sessions at W0 and W4. Participants were seated comfortably and instructed to relax without focusing on any particular thoughts. Data were recorded at a sampling frequency of 256 Hz using a 32-channel g. nautilus system (g.tec Medical Engineering GmbH, Austria). The 32 electrodes were distributed across the scalp based on the extended 10–20 international system using an elastic electrode cap, with the reference channel positioned on the right earlobe of each participant. We acquired EEG from participants with their eyes closed and open at rest for 5 min twice, respectively, totaling 20 min of resting-state data.

### Functional connectivity analysis using resting-state EEG

To analyze the changes in functional connectivity in the motor area before and after the intervention, the eyes-closed resting-state EEG data was first preprocessed as follows: The raw EEG data were bandpass-filtered using a 3rd-order Butterworth filter with cutoff frequencies of 1 and 50 Hz, and then segmented into 1-s epochs without overlaps [17]. Epochs containing significant artifacts exceeding a ± 120 µV signal threshold were removed, and 30 artifact-free epochs were randomly selected for each participant. Among the 25 participants, one participant’s data from the MI-independent BCI group was excluded due to insufficient artifact-free epochs (< 30). Thus, pre-processed EEG data from 24 patients (MI-contingent feedback BCI group = 12, MI-independent feedback BCI group = 12) were utilized for functional connectivity analysis. EEG channels were inverted for individuals with right-hemisphere lesions to ensure consistent data analysis across patients, aligning the lesion consistently over the left hemisphere in all the participants. For example, electrode C3* was assigned to cover the affected hemisphere, while electrode C4* was designated for the unaffected hemisphere. Partial directed coherence (PDC) [18] is a statistical measure used to determine the direction and strength of interactions between time series in the frequency domain, particularly in the context of neural data analysis such as EEG. PDC is derived from multivariate autoregressive (MVAR) models, which allows it to identify the direct influence of one variable on another while controlling for the effect of all other variables in the system. In this study, Partial directed coherence (PDC), a representative, effective functional connectivity measure, was employed to assess changes in directed functional connectivity pre- and post-intervention [9, 19]. PDC was calculated for each of the 30 pre-processed EEG epochs using a 6th-order multivariate autoregressive model implemented in the Hermes Matlab toolbox [20]. The µ (10–12 Hz) and β (18–24 Hz) bands, which are the frequencies most relevant to motor control. were used for PDC calculation [21]. Subsequently, PDC values were normalized to the range of 0–1 and averaged across all 30 epochs for each participant.

### Transcranial magnetic stimulation (TMS)-induced motor evoked potential (MEP)

Cortical excitability was measured using a TMS (MagPro stimulator, MagVenture, Lucernemarken, Denmark) at W0 and W4. Participants were seated comfortably in a reclining armchair with their hands pronated on a cushion. We used a figure-of-8 coil to stimulate the motor cortex with the coil handle oriented 45° posterior to the midline to ensure the electromagnetic current flowed perpendicularly to the central sulcus [22]. Electromyographic signals were recorded using an active surface electrode placed on the contralateral first dorsal interosseous muscle, while reference and ground electrodes were positioned on the index finger proximal interphalangeal joint and over the wrist, respectively. The optimal scalp location (hotspot) was determined as the site eliciting the largest MEP amplitude with the lowest stimulation intensity [23].

We examined cortical excitability using MEPs and resting motor threshold (RMT). The RMT (%) was the lowest stimulator intensity that could elicit MEPs with an amplitude of at least 50 microvolts in at least five out of 10 consecutive trials [23]. MEP amplitude was evaluated at 120% of the TMS intensity necessary to elicit RMT, and we measured the average peak-to-peak amplitudes of MEP from 10 consecutive MEP sweeps.

### Statistical analysis

We employed a linear mixed model analysis for repeated measurements to compare intervention effects within and between groups across all time points. The statistical model included the outcomes as the dependent variable, with group (between-subject factor; MI-contingent feedback BCI or MI-independent feedback BCI), time (within-subject factor), group × time interactions, and baseline value of the outcome as fixed effects. Each participant’s intercept was considered a random intercept in the model. Group × time interactions were utilized to estimate intervention effects at all time points, while within-group time effects were assessed in each group. Results are presented as actual values and mean adjusted differences in outcomes between the two groups, with 95% confidence intervals (CI). Additionally, we examined correlations between primary outcomes (MRC-WE and AROM-WE) and PDC from premotor to motor in the µ and β frequency bands using repeated measures correlation (rmcorr) to identify linear relationships in paired data collected through repeated measurements. The analysis employed R version 4.3.2 (http://www.r-project.org) using the lme4 and rmcorr package [24, 25]. MEP data were not statistically analyzed because MEP was observed in only five participants.

## Results

### Clinical outcomes

This study included a total of 27 participants with chronic stroke. Among these, two participants in the MI-contingent feedback BCI group dropped out because of transfer to another hospital. Consequently, 25 participants (12 patients in the MI-contingent feedback BCI group, 13 patients in the MI-independent feedback BCI group) were examined (Fig. 1). The participants’ baseline demographic and clinical characteristics are presented in Table 1, revealing no significant differences between the groups.

**Fig. 4:**
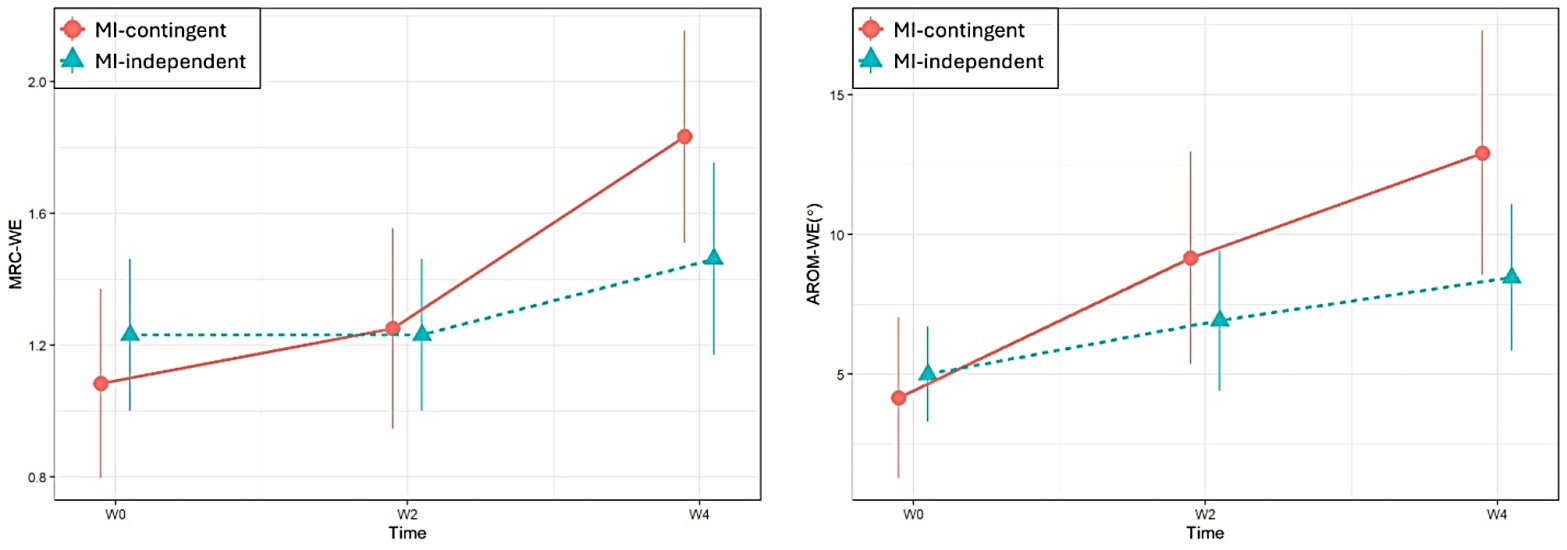
Changes in MRC-WE and AROM-WE from W0 to W4. MRC-WE, Medical Research Council scale score for muscle strength in the wrist extensor; AROM-WE, active range of motion in wrist extension

Figure 4; Table 2 illustrate the changes in primary outcomes (MRC-WE and AROM-WE) from W0 to W4. The MI-contingent feedback BCI group exhibited significant improvements in MRC-WE at W4, as evidenced by the interaction effect over time, in comparison to the MI-independent feedback BCI group (*p* = 0.036). Specifically, the MRC-WE score was 0.52 higher in the MI-contingent feedback BCI group than in the MI-independent feedback BCI group (95% CI: 0.03–1.00). Moreover, significant improvements in MRC-WE and AROM-WE were observed solely in the MI-contingent feedback BCI group at W4 compared to W0 (*p* = 0.002; *p* = 0.019, respectively).

**Table 1:** Participants’ demographic data

	MI-contingent BCI group (*n* = 12)		MI-independent BCI group (*n* = 13)
Sex (Male/Female)	10/2		9 /4
Age	49.0 ± 16.9^a^		46.0 ± 12.8^a^
Affected brain lesion (right/left)	6/6	10/3	
Type of stroke (hemorrhage/infarction)	7/5	9/4	
Onset time (month)	18.2 ± 16.2^a^	22.2 ± 23.4^a^	
FMA	29.9 ± 12.0^a^	33.5 ± 11.1^a^	
MRC-WE	1.1 ± 1.0^a^	1.2 ± 0.8^a^	
Modified Ashworth wrist flexor	0.4 ± 0.8^a^	0.1 ± 0.3^a^	
MEP (positive/negative)	3/9	2/11	

^a^Mean ± Standard deviation

BCI, brain-computer interface; FMA, Fugl-Meyer assessment; MRC-WE, muscle strength in the wrist extensor; MEP, motor evoked potential

Table 2 presents secondary outcome measures with estimated mean differences between groups across time from a linear mixed model. No time × group interaction was noted for FMA (FMA-total, FMA-distal, and FMA-hand). However, the MI-independent feedback BCI group demonstrated significant improvements in FMA-total (*p* = 0.005), FMA-distal (*p* = 0.020), and FMA-hand (*p* = 0.009) at W4 compared to W0, whereas FMA did not improve within the MI-contingent feedback BCI group. Additionally, there were no significant time × group interaction or main effects of time for variables other than those described above: SIS, MAS-WF, and BBT. Furthermore, the t-test for independent samples comparing the average MI accuracy between the two groups showed no statistically significant difference (*p* = 0.486). The experimental group had a mean MI accuracy of 71.23 ± 9.04, while the control group had a mean MI accuracy of 68.20 ± 12.30. No adverse events were reported.

**Table 2:** Linear mixed model analysis of outcomes

	unadjusted mean ± SD								adjusted difference	(95% CI)	*p*-value^1^
	MI-contingent BCI			MI-independent BCI							
MRC-WE											
W0	1.083	±	0.996	1.230		±	0.832				
W2	1.250	±	1.055	1.230		±	0.832		0.17	-0.32–0.65	0.495
W4	1.833	±	1.114	1.461		±	1.050		0.52	0.03–1.00	**0.036**
(W2) p-value^2^	0.669			1.000							
(W4) p-value^2^	**0.002**			0.294							
AROM-WE											
W0	4.166	±	9.962	5.000	±			6.123			
W2	9.166	±	13.113	6.923	±			9.022	3.08	-4.27–10.42	0.406
W4	12.916	±	15.144	8.461	±			9.439	5.29	-2.06–12.63	0.155
(W2) p-value^2^	0.231			0.672							
(W4) p-value^2^	**0.019**			0.290							
FMA-total											
W0	29.917	±	11.950	33.538	±			11.095			
W2	31.916	±	12.964	33.923	±			11.243	1.62	-1.03–4.26	0.227
W4	31.333	±	14.047	36.307	±			11.397	-1.35	-4.00–1.29	0.311
(W2) p-value^2^	0.175			0.879							
(W4) p-value^2^	0.402			**0.005**							
FMA-distal											
W0	4.333	±	5.123	6.308		±	4.785				
W2	5.250	±	4.845	6.846		±	5.129		0.38	-1.40–2.16	0.673
W4	5.416	±	5.664	8.076		±	5.529		-0.69	-2.47–1.10	0.445
(W2) p-value^2^	0.357			0.655							
(W4) p-value^2^	0.244			**0.020**							
FMA-wrist											
W0	0.833	±	2.588	1.462		±	2.025				
W2	1.333	±	2.708	1.538		±	1.853		0.42	-0.49–1.33	0.357
W4	1.250	±	2.895	2.153		±	2.409		-0.28	-1.19–0.64	0.548
(W2) p-value^2^	0.156			0.976							
(W4) p-value^2^	0.266			0.163							
FMA-hand											
W0	3.500	±	2.939	4.846		±	4.120				
W2	3.917	±	2.644	5.307		±	4.190		-0.04	-1.27–1.18	0.942
W4	4.166	±	3.537	5.922		±	4.251		-0.41	-1.63–0.81	0.505
(W2) p-value^2^	0.712			0.359							
(W4) p-value^2^	0.428			**0.009**							
MAS-WF											
W0	1.333	±	1.230	5.692		±	7.674				
W2	1.416	±	0.900	5.538		±	7.456		0.31	-0.36–0.98	0.352
W4	1.666	±	1.073	6.153		±	8.214		0.56	-0.11–1.23	0.097
(W2) p-value^2^	0.958			0.354							
(W4) p-value^2^	0.519			0.354							
BBT											
W0	2.083	±	5.107	5.692		±	7.674				
W2	2.666	±	6.242	5.538		±	7.456		0.74	-0.52–1.99	0.246
W4	2.916	±	6.459	6.153		±	8.214		0.37	-0.89–1.63	0.557
(W2) p-value^2^	0.229			0.952							
(W4) p-value^2^	0.060			0.647							
SIS-hand function											
W0	12.917	±	23.496	16.153		±	19.273				
W2	12.916	±	29.034	17.692		±	17.513		-1.54	-14.69–11.61	0.816
W4	5.833	±	11.645	18.076		±	20.056		-9.01	-22.15–4.14	0.176
(W2) p-value^2^	1.000			0.927							
(W4) p-value^2^	0.391			0.889							
SIS-ADL/IADL											
W0	68.333	±	20.871	70.000		±	18.568				
W2	73.125	±	17.060	74.038		±	12.891		0.75	-11.58–13.08	0.903
W4	68.333	±	18.163	65.192		±	18.887		4.81	-7.52–17.14	0.439
(W2) p-value^2^	0.480			0.657							
(W4) p-value^2^	1.000			0.554							
SIS-social participation											
W0	45.325	±	31.000	39.915		±	19.046				
W2	54.966	±	24.941	49.061		±	23.751		0.50	-20.21–21.20	0.961
W4	56.266	±	27.703	38.953		±	26.549		11.90	-8.80–32.61	0.239
(W2) p-value^2^	0.372			0.415							
(W4) p-value^2^	0.284			0.989							

^1^ p-values for group × time interactions

^2^ p-values for time effects

BCI, brain-computer interface; FMA, Fugl-Meyer assessment; MAS, modified Ashworth scale; WF, wrist flexor

### Neurophysiological outcomes

**Fig. 5:**
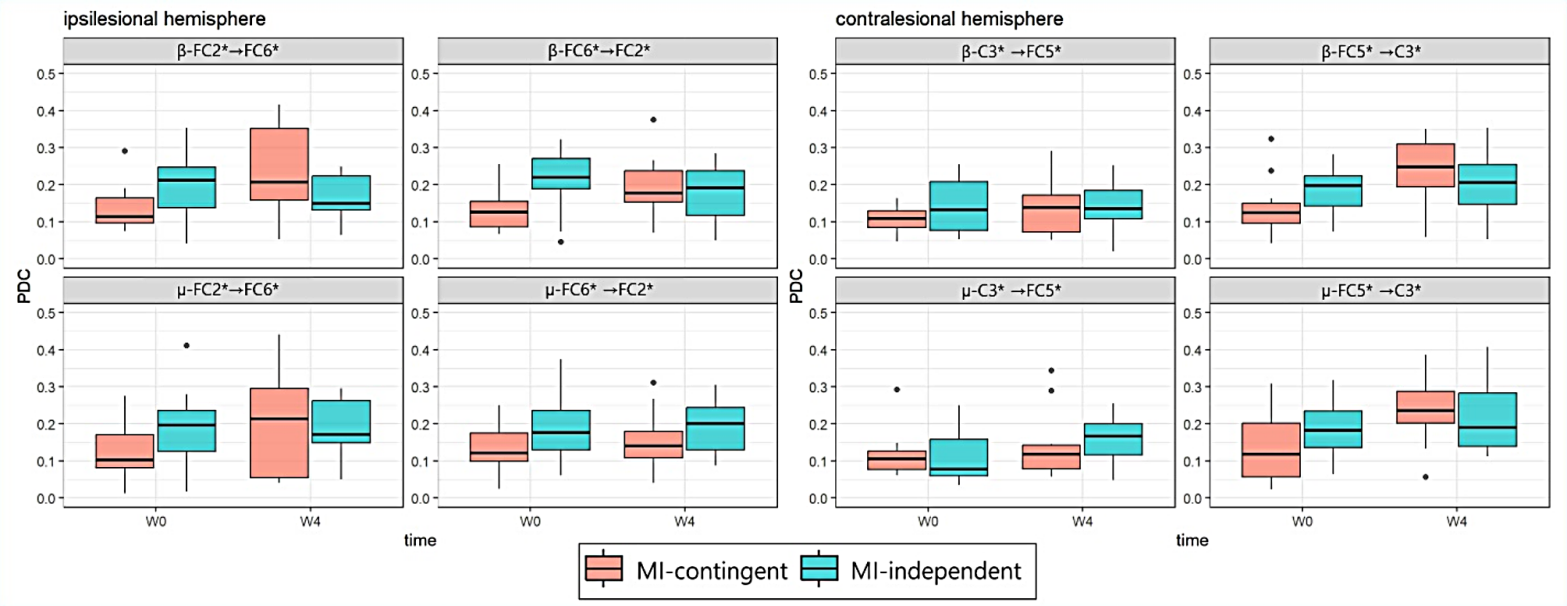
Significant PDC values for the ipsilesional hemisphere and contralateral hemisphere in both groups. PDC, partial directed coherence

**Fig. 6:**
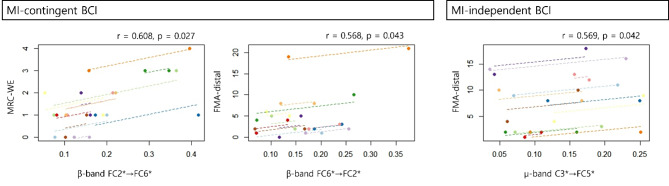
Repeated measures correlation analysis of the associations of changes in β-band and µ-band with MRC-WE and FMA-distal in the two groups. MRC-WE, Medical Research Council (MRC) scale score for muscle strength in the wrist extensor; FMA, Fugl-Meyer assessment

Figure 5 illustrates the changes in PDC values in both the groups (Additional File 1). Significant time × group interactions were observed for effective connectivity in the ipsilesional premotor area for the β frequency band (FC2*→FC6* PDC: *p* = 0.005 and FC6*→FC2* PDC: *p* = 0.014). The MI-contingent feedback BCI group exhibited enhancement in the ipsilesional premotor area (β-band FC2*→FC6* PDC: *p* = 0.016) and the contralesional premotor area to the motor area (µ-band FC5*→C3* PDC: *p* = 0.017; β-band FC5*→C3*: *p* = 0.012). In contrast, the MI-independent feedback BCI group did not display significant changes. Additionally, a repeated measures correlation analysis revealed a significant correlation between the change of β-band FC2*→FC6* PDC value and MRC-WE (*r* = 0.608, *p* = 0.027), and between the β-band FC6*→FC2* PDC value and FMA-distal (*r* = 0.568, *p* = 0.043) in the MI-contingent feedback BCI group (Fig. 6). Moreover, a repeated measures correlation analysis indicated a significant correlation between the change of µ-band C3*→FC5* PDC value and FMA-distal (*r* = 0.569, *p* = 0.042) in the MI-independent feedback BCI group.

**Fig. 7:**
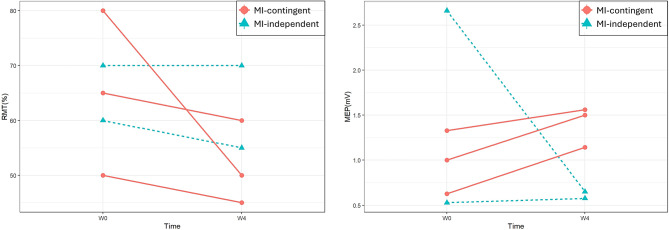
Individual RMT and MEP data for the two groups. RMT, resting motor threshold; MEP, motor evoked potential

Figure 7 illustrates individual data for five participants (three from the MI-contingent feedback BCI group and two from the MI-independent feedback BCI group) who exhibited MEP. RMT data indicated a decrement in four participants, while one participant in the MI-independent feedback BCI group showed no change after the intervention. MEP data revealed improvement in four participants, but one participant in the MI-independent feedback BCI group exhibited a decrement in MEP after intervention.

## Discussion

In this double-blinded randomized controlled trial, we observed that the MI-contingent BCI group outperformed the MI-independent BCI group in primary outcomes (MRC-WE) among persons with chronic stroke. The MI-contingent BCI and MI-independent BCI groups improved from 1.08 to 1.83 and from 1.23 to 1.46, respectively, after 4 weeks of BCI intervention. This superiority of MI-contingent BCI over MI-independent BCI was consistent with a previous study, wherein 6 weeks of FES-based BCI improved MRC-WE from 1.43 to 2.57 in the MI-contingent BCI group and from 1.31 to 1.62 in the MI-independent BCI group [9]. AROM-WE, another primary outcome, also improved in the MI-contingent BCI group, but not in the MI-independent BCI group.

These clinical improvements coincided with changes in functional connectivity. Effective connectivity in the premotor area of the affected hemisphere, as well as connectivity from the premotor area to the motor area in the unaffected hemisphere, improved significantly only in the MI-contingent BCI group. Specifically, a significant time x group interaction in PDC-based functional connectivity was observed in the ipsilesional premotor area in the MI-contingent BCI group, but not in the MI-independent BCI group. Additionally, functional connectivity enhancements were evident in the ipsilesional premotor area and from the contralesional premotor to the motor area solely in the MI-contingent BCI group. This enhancement in ipsilesional functional connectivity indicates an upsurge in motor neuron excitability corresponding to desired movements, eliciting sufficient voluntary action potential for motor execution, aligning with previous findings showing robust desynchronized activity in the ipsilesional hemisphere with MI-based BCI [5, 9]. Furthermore, improvements in contralesional hemisphere connectivity throughout the MI-contingent BCI suggest that BCI facilitated complex bilateral brain activity contributing to motor recovery in persons with chronic stroke [26–28]. These improvements, observed exclusively in the MI-contingent BCI group, suggest that real-BCI facilitated neuronal plasticity, as functional connectivity derived from PDC could capture causal relationships following neurological interventions [5, 29].

Crucially, the correlation between the change in ipsilesional connectivity and the improvement in MRC-WE and FMA-distal within the MI-contingent BCI group confirms that real-BCI induces plastic changes in the brain, facilitating functional enhancements, consistent with prior findings where BCI-FES induced functional improvements associated with brain network connectivity [9, 30]. Similarly, the consistent improvement observed in RMT and MEP findings in the MI-contingent BCI group contrasts with the either deteriorated or unchanged outcomes in the MI-independent BCI group. While this suggests that MI-contingent BCI may affect the corticospinal tract, caution is warranted due to the limited availability of MEP data, which were only accessible for five participants. Thus, when considering the neurophysiological findings, it can be inferred that MI-contingent BCI drives clinical improvements by initiating brain changes and subsequent modifications in the motor pathway from the brain to the end-effector. Previous studies have indicated that contingency is critical to improve the effects of interventions such as FES or robots [8, 9]. MI-contingent real-time feedback facilitates the acquisition of the motor strategy and promotes long-term retention of the motor task [31]. Therefore, with an MI-contingent BCI, individuals may develop the ability to modulate the brain’s oscillatory activity triggered by MI or motor attempts. This skill is honed through immediate and precise somatosensory feedback, potentially establishing a new sensorimotor loop by strengthening the associative connection between MI and feedback, consistent with Hebbian plasticity principles.

Meanwhile, our findings regarding the FMA differed from previous studies where BCI significantly influenced the outcomes of FMA [7–9]. In our MI-contingent BCI group, we did not observe statistically significant improvements, whereas the MI-independent BCI group demonstrated significant within-group enhancements in the FMA. This disparity may stem from differences in the feedback mechanism. Unlike previous studies in which the MI-independent BCI did not provide feedback upon MI failure, our MI-independent BCI group consistently received FES regardless of MI success. Consequently, the MI-independent BCI group underwent more FES repetitions, as they always received stimulation regardless of MI accuracy, unlike the MI-contingent BCI group, which received FES only upon successful MI. Additionally, our MI-independent BCI group may have experienced a higher magnitude of the placebo effect compared to the sham-BCI groups in previous studies, as participants may have erroneously believed they were receiving real BCI intervention. We believe that these findings also influenced the significant correlation observed between the change in µ-band C3*→FC5* PDC value and FMA-distal in the MI-independent feedback BCI group. Furthermore, certain FMA items, such as mass flexion and grasp, were irrelevant to our BCI interventions.

Our BCI system utilized FES as a feedback mechanism. Recent systematic reviews have highlighted that only FES triggered by BCI significantly impacts motor function, unlike robot-assisted or virtual visual feedback interventions [32, 33]. FES delivers sensory feedback regarding joint position and muscle tension, thereby refining participants’ movements within the feedback loop and enhancing their awareness of movement, ultimately improving cortical excitability [34]. Moreover, our FES targeted the wrist extensors, simplifying task comprehension for participants and facilitating learning through repetitive movements. This simple approach complements a simple bottom-up strategy, effectively establishing a meaningful closed loop when combined with motor imagination, which represents a top-down approach.

Previous studies have reported that one of the effects of BCI is to improve the quality of life (QoL) [5, 35, 36]. Sinha et al. [35] demonstrated statistically significant improvements in specific indicators of SIS following BCI application in persons with stroke. Similarly, the present study also predicted the effectiveness of BCI intervention using SIS, which was used as a QoL indicator. However, statistically significant changes were not observed in both the MI-contingent BCI group and the MI-independent BCI group. It is a clinically known fact that improvements in motor function do not always mean improvements in independence and performance of ADL. Zanona et al. [37] suggested that functional improvement does not necessarily lead to improvements in ADL. Moreover, a study showed that FMA improved in persons with chronic stroke without any changes in ADL [38]. Therefore, whether improvements in upper arm movements can be beneficial for performing ADL remains questionable. A randomized controlled trial applying BCI for persons with upper limb stroke showed that outcomes measured by SIS may vary depending on the stroke severity and chronicity [39]. Among the stroke subjects in our study, the experimental and control groups had average onset times of 18.2 and 22.2 months, respectively; thus, the target population was persons with chronic stroke. Subsequent research may need to consider stroke onset and severity when assessing QoL. Briefly, the improvement in wrist function did not translate into improvements in other outcomes. The intervention in our study did not involve task-specific functional training which might explain the observed outcomes. Many participants had severe impairments and limited distal function and, despite functional improvements, did thus not show improvements in FMA, BBT, or QoL measures.

This study has some limitations. First, the sample size was not determined based on prior research findings. This challenge arose due to the variability in BCI systems, each possessing unique characteristics in acquiring brain signals and delivering neurofeedback. Second, our study exclusively enrolled persons with chronic stroke, overlooking the potential plasticity observed in the early phases of stroke. Future investigations should extend to include patients in the early stages of stroke to capture the full spectrum of neuroplastic changes. Third, we were unable to compare the effects of BCI based on stroke lesion location and size because the sample size was too small to conduct meaningful subgroup analysis. Future research should continue to investigate this aspect. Finally, participants in our study exhibited severe impairments, hindering the attainment of clinically significant functional improvements and reliable MEP data crucial for understanding the function of the descending corticospinal tract. Therefore, future studies should consider recruiting patients with mild to moderate stroke severity to broaden the applicability of BCI interventions and deepen our understanding of their underlying mechanisms.

## Conclusions

This study highlights the differential impacts of MI-contingent and MI-independent BCI training on upper limb rehabilitation for individuals with chronic stroke. MI-contingent BCI demonstrated significant improvements in wrist extensor function, specifically in MRC-WE and AROM-WE, highlighting its efficacy in targeting task-specific impairments. On the other hand, the MI-independent group showed superior outcomes in improving motor control, coordination, or the ability to perform specific movements, as evidenced by larger decreases in FMA scores. We believe that these findings were also influenced by the effects of FES and repetitive training. Importantly, the observed changes in EEG patterns differed between the two interventions, suggesting that both were effective, albeit through distinct mechanisms.

Additionally, it is important to consider the role of chronicity in these outcomes, as the chronic stage of stroke recovery may limit neural plasticity and functional reorganization. To address this, ongoing research is investigating the efficacy of BCI-based interventions in patients with subacute stroke, where the potential for recovery may be greater. These studies are expected to provide deeper insights into the fundamental mechanisms underlying BCI effectiveness and its potential to optimize rehabilitation strategies during various stages of stroke recovery.

## Electronic supplementary material

Below is the link to the electronic supplementary material.

Supplementary Material 1

## Data Availability

The datasets analyzed during the current study are available from the corresponding author on reasonable request.

## Abbreviations

BBT: Box and Block Test
BCI: Brain-computer interface
CI: Confidence intervals
CNS: Central nervous system
FES: Functional electrical stimulation
FMA: Fugl-Meyer assessment
HRQoL: Health-related quality of life
MAS: Modified Ashworth scale
MEP: Motor evoked potential
MI: Motor imagery
MRC: Medical Research Council
PDC: Partial directed coherence
RMT: Resting motor threshold
SIS: Stroke Impact Scale
TMS: Transcranial magnetic stimulation
