# Supplementary material for: Efficacy of brain-computer interface training with motor imagery-contingent feedback in improving upper limb function and neuroplasticity among persons with chronic stroke: a double-blinded, parallel-group, randomized controlled trial
Source: J Neuroeng Rehabil. 2025 Jan 6;22:1. doi: 10.1186/s12984-024-01535-2 (PMC11702034; doi:10.1186/s12984-024-01535-2)
Supplement: Supplementary file 1 — Supplementary Material 1 [file 12984_2024_1535_MOESM1_ESM.docx]

**Supplementary Table 1**. Linear mixed model analysis of secondary outcomes

|  | Unadjusted mean ± SD | | | | | | Adjusted difference | (95% CI) | p-value^1^ |
| --- | --- | --- | --- | --- | --- | --- | --- | --- | --- |
|  | MI-contingent BCI | | | MI-independent BCI | | |  |  |  |
| β-band FC2*→FC6* PDC value | | | | | | | | | |
| W0 | 0.137 | ± | 0.062 | 0.196 | ± | 0.088 |  |  |  |
| W4 | 0.236 | ± | 0.117 | 0.165 | ± | 0.055 | 0.13 | 0.04–0.22 | 0.005 |
| (W4) p-value^2^ | 0.0157 | | | 0.3072 | | |  |  |  |
| β-band FC6*→FC2* PDC value | | | | | | | | | |
| W0 | 0.131 | ± | 0.055 | 0.213 | ± | 0.086 |  |  |  |
| W4 | 0.193 | ± | 0.081 | 0.171 | ± | 0.082 | 0.1 | 0.02–0.19 | 0.014 |
| (W4) p-value^2^ | 0.051 | | | 0.232 | | |  |  |  |
| μ-band FC2*→FC6* PDC value | | | | | | | | | |
| W0 | 0.125 | ± | 0.072 | 0.192 | ± | 0.101 |  |  |  |
| W4 | 0.207 | ± | 0.143 | 0.187 | ± | 0.081 | 0.09 | -0.02–0.19 | 0.094 |
| (W4) p-value^2^ | 0.065 | | | 0.8882 | | |  |  |  |
| μ-band FC6*→FC2* PDC value | | | | | | | | | |
| W0 | 0.132 | ± | 0.060 | 0.188 | ± | 0.087 |  |  |  |
| W4 | 0.153 | ± | 0.082 | 0.192 | ± | 0.073 | 0.02 | -0.06–0.09 | 0.667 |
| (W4) p-value^2^ | 0.471 | | | 0.903 | | |  |  |  |
| β-band FC5*→C3* PDC value | | | | | | | | | |
| W0 | 0.137 | ± | 0.080 | 0.186 | ± | 0.063 |  |  |  |
| W4 | 0.234 | ± | 0.093 | 0.202 | ± | 0.094 | 0.08 | -0.03–0.16 | 0.095 |
| (W4) p-value^2^ | 0.0119 | | | 0.626 | | |  |  |  |
| β-band C3*→ FC5* PDC value | | | | | | | | | |
| W0 | 0.106 | ± | 0.035 | 0.142 | ± | 0.074 |  |  |  |
| W4 | 0.132 | ± | 0.070 | 0.141 | ± | 0.063 | 0.03 | -0.04–0.10 | 0.42 |
| (W4) p-value^2^ | 0.256 | | | 0.962 | | |  |  |  |
| μ-band FC5*→C3* PDC value | | | | | | | | | |
| W0 | 0.137 | ± | 0.094 | 0.188 | ± | 0.072 |  |  |  |
| W4 | 0.233 | ± | 0.087 | 0.115 | ± | 0.076 | 0.06 | -0.03–0.16 | 0.192 |
| (W4) p-value^2^ | 0.0166 | | | 0.3664 | | |  |  |  |
| μ-band C3*→FC5* PDC value | | | | | | | | | |
| W0 | 0.116 | ± | 0.063 | 0.220 | ± | 0.095 |  |  |  |
| W4 | 0.140 | ± | 0.089 | 0.162 | ± | 0.060 | -0.02 | -0.10–0.06 | 0.578 |
| (W4) p-value^2^ | 0.445 | | | 0.109 | | |  |  |  |

^1^ p-values for group × time interactions.

^2^ p-values for time effects.

PDC, partial directed coherence; BCI, brain-computer interface
